# YBX-1 mediated sorting of miR-133 into hypoxia/reoxygenation-induced EPC-derived exosomes to increase fibroblast angiogenesis and MEndoT

**DOI:** 10.1186/s13287-019-1377-8

**Published:** 2019-08-23

**Authors:** Fengxia Lin, Zhicong Zeng, Yinzhi Song, Liang Li, Zijun Wu, Xiaoduo Zhang, Zhiwen Li, Xiao Ke, Xun Hu

**Affiliations:** 1 Department of Cardiology, Shenzhen Bao’an Traditional Chinese Medicine Hospital Group, The Affiliated Hospital of Guangzhou University of Chinese Medicine, Shenzhen, 518133 China; 2 Graduate School, Guangzhou University of Chinese Medicine, Guangzhou, 510405 China; 3 Department of Cardiology, Fuwai Hospital, Chinese Academy of Medical Sciences, Shenzhen, 518057 Guangdong China; 4 Shenzhen University School of Medicine & Shenzhen University Health Science Center, No. 12, Langshan Road, Nanshan District, Shenzhen, 518057 Guangdong China; 5 Department of Cardiology, The First Affiliated Hospital, Sun Yat-Sen University, Guangzhou, 510080 Guangdong China; 6 Key Laboratory on Assisted Circulation, Ministry of Health, Guangzhou, 510080 Guangdong China

**Keywords:** Endothelial progenitor cell, Myocardial fibrosis, miR-133, Exosome, Y box binding protein 1

## Abstract

**Background:**

Myocardial fibrosis is a common pathophysiological change in cardiovascular disease, which can cause cardiac dysfunction and even sudden death. Excessively activated fibroblasts proliferate and secret excessive extracellular matrix (ECM) components, resulting in normal cardiac structural damage and cardiac fibrosis. We previously found that human endothelial progenitor cell (EPC)-derived exosomes, after hypoxia/reoxygenation (H/R) induction, could significantly increase the mesenchymal-endothelial transition (MEndoT) compared to normal culture EPC-derived exosomes. Exosomes have been shown to carry different nucleic acids, including microRNAs. However, the effects of microRNAs in EPC-derived exosomes on MEndoT and myocardial fibrosis remain unknown.

**Methods:**

EPCs were isolated from human peripheral blood, and fibroblasts were isolated from rat hearts, then transfected with miR-133 inhibitor, si-YBX-1, and ov-YBX-1 into EPCs. After H/R induction for 48 h, isolation and characterization of exosomes derived from human EPCs were performed. Finally, fibroblasts were treated by exosome at 48 h. The expression of miR-133 was measured by qRT-PCR; YBX-1 expression was measured by qRT-PCR and western blot. Angiopoiesis was measured by tube formation assay. Endothelial markers and fibrosis markers were measured by western blot.

**Results:**

H/R treatment promoted miR-133 expression in EPCs and EPC-derived exosomes. miR-133 could be incorporated into exosomes and transmitted to cardiac fibroblasts, increasing the angiogenesis and MEndoT of cardiac fibroblasts. miR-133 silencing in H/R-induced EPCs could inhibit miR-133 expression in EPCs and EPCs-derived exosomes. miR-133 silencing in H/R-induced EPCs could inhibit the angiogenesis and MEndoT of cardiac fibroblasts and reverse the effect of H/R treatment. Additionally, miR-133 was specially sorted into H/R-induced EPC-derived exosomes via YBX-1. YBX-1 silencing inhibited miR-133 transfer and reduced fibroblast angiogenesis and MEndoT.

**Conclusion:**

miR-133 was specially sorted into H/R-induced EPC-derived exosomes via YBX-1 to increase fibroblast angiogenesis and MEndoT.

## Background

Myocardial fibrosis is a common pathophysiological change in cardiovascular disease, which can cause cardiac dysfunction and even sudden death [1]. It is one of the important causes of death in patients with cardiovascular disease. Therefore, reversing myocardial fibrosis is an important goal in the treatment of cardiovascular disease. Fibroblasts are key effector molecules of cardiac fibrosis [2]. Cardiac fibroblasts play an important role in maintaining cardiac structure and function by synthesizing collagen I, collagen III, fibronectin, and other extracellular matrix components. Under pathological conditions, fibroblasts proliferate and activate excessive secretion of extracellular matrix components, resulting in normal cardiac structural damage and cardiac fibrosis. Cardiac fibroblasts were once thought to be terminally differentiated cells. However, recent studies have shown that chromatin-modifying agents could activate Wnt signaling to transform human adult dermal fibroblast cells to OCT4+ and VEGFR-2+ capillary tube-forming cells [3]. When pathological damage occurs in the heart, fibroblasts undergo a mesenchymal-endothelial transition (MEndoT) to obtain endothelial cell-like functions and participate in angiogenesis in the cardiac injury area, which is a novel antifibrotic strategy to alleviate myocardial fibrosis [4]. However, the mechanism underlying the transformation of cardiac fibroblasts into endothelial cells during pathological heart damage remains unclear.

Previously, we found that hypoxia/reoxygenation (H/R)-induced human endothelial progenitor cell (EPC)-derived exosomes increased proliferation and angiogenesis of cardiac fibroblasts by promoting MEndoT [5]. Exosomes are small membrane vesicles that mediate intercellular signal transduction [6]. Previous studies have shown that EPC-derived exosomes can promote angiogenesis and reduce cardiomyocyte hypertrophy and apoptosis [7–9]. Furthermore, exosomes had been shown to carry different nucleic acids, including microRNAs (miRNAs), which are acquired by recipient cells to regulate their own fate [10, 11]. Previous studies found that miRNAs in exosomes from endothelial progenitor cells improved outcomes of patients with cardiovascular disease [12] and lipopolysaccharide-induced acute lung injury [13] and also of a murine model of sepsis [12].

miRNAs significantly regulate cell growth and metabolism through post-transcriptional inhibition of gene expression and play important roles in myocardial fibrosis [14–16]. miR-181a and miRNA-221/222 regulated cardiac fibroblast activation to increase deposition of extracellular matrix components and promote myocardial fibrosis [17, 18]. miR-378 in exosomes, which is secreted from cardiomyocytes, inhibited excessive cardiac fibrosis [19]. Further, miR-217 in exosomes, which is secreted from cardiomyocytes, promoted cardiac fibrosis processes, cardiac hypertrophy, and cardiac fibrosis processes [20]. However, the effects of miRNA in EPC-derived exosomes on MEndoT and myocardial fibrosis remain unknown.

In our previous study, we found that EPC-derived exosomes, after H/R induction, could significantly increase MEndoT compared with normal culture EPC-derived exosomes [5]. In this study, we collected exosomes derived from normal and H/R-cultured EPCs and measured miRNA expression using a miRNA array. Additionally, we investigated the effect of miRNAs in EPC-derived exosomes on angiogenesis and MEndoT. Finally, we examined the mechanism of miRNA assembly into exosomes.

## Materials and methods

### Isolation, identification, and culture of EPCs and fibroblasts

EPCs were isolated from human peripheral blood, and fibroblasts were isolated from rat hearts which are identified by immunofluorescence and flow cytometry as previously described [5]. Isolated EPC cells were placed into a 25-cm^2^ culture bottle and cultured in Dulbecco’s modified Eagle’s medium (DMEM; Gibco) containing 10% fetal bovine serum, which was centrifuged in advance by density gradient centrifugation to remove existing exosomes (Gibco), in a 5% CO_2_ humidified environment at 37 °C. The medium was changed every 3 days. Late passage cells (p3) were used in subsequent experiments. Isolated fibroblasts were suspended in DMEM (Gibco) with 10% FBS (Gibco). After 30 min, cells that were weakly attached or unattached were discarded. Cells were seeded onto 35-mm plates (1 × 10^5^ cells/plate) for 3 days.

### H/R treatment

EPCs were grown to 80% confluence and quiesced for 12 h. Plated cells were subjected to normoxic or hypoxic conditions for 12 h. To generate hypoxic conditions, cells were transferred to an incubation chamber (Billups-Rothenberg MIC-101) and flushed with hypoxic gas mixture (95% N_2_, 5% CO_2_). Subsequently, the cells were cultured in normoxic conditions for 48 h.

### Isolation and characterization of exosomes derived from human EPCs

Exosomes derived from H/R-treated EPCs were isolated and identified as previously described [5]. Following H/R treatment of EPCs, the original medium was replaced with fresh exosomal-free serum medium, and the cells were cultured for 24 h. Culture media of EPCs were collected and centrifuged at 3000 *g* for 30 min and 100,000 *g* for 90 min at 4 °C to remove dead cells and cellular debris by Optima Ultracentrifuge (Beckman Coulter). The medium was mixed with 0.5 mL of Total Exosome Isolation reagent (GENESEED, Guangzhou, China), centrifuged at 10,000*g* for 1 h at 4 °C to obtain exosomes. Exosome morphology was visualized using a transmission electron microscope (Hitachi H-7650; Japan), and images were taken with a digital camera (Olympus). Surface proteins (CD63, TSG101, and HSP70) on the exosomes were detected by western blotting. Finally, EPC-derived exosomes were added to the fibroblast culture medium.

### Apoptosis and senescence assay

The apoptosis of H/R-treated EPCs was measured by the Annexin V-fluorescein isothiocyanate (FITC) Apoptosis Detection Kit (Keygentec, Nangjing, China). The senescence of H/R-treated EPCs was measured by Senescence β-galactosidase staining kit (Beyotime, Shanghai, China).

### miRNA profiling

Exosomal RNA was extracted using the TRIzol reagent (Life Technologies, Carlsbad, CA, USA). miRNA expression profile in exosomes was investigated by miRNA microarray analysis. Exosomal miRNAs were extended and hybridized with fluorescent-labeled biotin dyes on a Gene Chip miRNA 4.0 Array (Affymetrix, Cleveland, OH, USA). Following hybridization, the images were digitized and analyzed using a laser scanner interfaced with ArrayPro image analysis software (Media Cybernetics, Silver Spring, MD, USA). Data were analyzed by first subtracting the background, followed by normalizing the signals using a LOWESS filter (locally weighted regression) [21]. The differentially expressed miRNAs were defined using the ratio of detected signals log2-fold changes [log2(mTLE-HS/control)], and the Student’s *t* test was used to calculate *P* values. Those with a log2 ratio > 1.0 or ≤ − 1.0 and *P* values < 0.05 were considered as differentially expressed miRNAs. Cluster analysis based on the relative expression levels of miRNAs was also conducted.

### Quantitative real-time reverse transcription-polymerase chain reaction

Total RNA was isolated using the TRIzol reagent (Invitrogen, Carlsbad, CA, USA) and reverse transcribed. qRT-PCR was performed using the SYBR® Premix ExTaqTM II Kit (Takara, Dalian, China) to detect YBX-1 expression and the Mir-X miRNA qRT-PCR SYBR Kit (Clontech Laboratories, Inc., USA) to detect miR-133 on a 7500 Real-Time PCR System (Applied Biosystems, Foster City, CA, USA). The relative expression levels of mRNA and miRNA were calculated using the 2−ΔΔCT method. GAPDH and U6 served as reference genes, respectively. The primer sequences were as follows: YBX-1 forward, 5′-GATAAATTTAAACCTGAAAA-3′ and reverse, 5′-ATCTTGTTTCTATCTTCCAA-3′; miR-133 forward, 5′- ACACTCCAGCTGGGCAAAGTGCTTACAGTGC-3′ and reverse, 5′-CTCAACTGGTGTCGTGGA-3′; U6 forward, 5′-CTCGCTTCGGCAGCACA-3′ and reverse, 5′-AACGCTTCACGAATTTGCGT-3′; GAPDH forward, 5′-GCTCATTTGCAGGGGGGAG-3′ and reverse, 5′-GTTGGTGGTGCAGGAG GCA-3′. All reactions were performed in triplicate.

### Transfection

YBX-1 expression interference (si-YBX-1), si-negative control (si-NC), miR-133 inhibitor, negative control inhibitor (NC inhibitor), miR-133 mimic, and NC mimic were purchased from RiboBio (Guangzhou, China). The open reading frame of YBX was synthesized and linked into pcDNA 3.1 (ov-YBX-1), and pcDNA 3.1 served as a negative control (ov-NC). EPCs (2 × 10^5^ cells/well) were transfected with 50 nM miR-133 inhibitor, 50 nM NC inhibitor, 50 nM si-YBX-1, 50 nM si-NC, 1 μg/μL ov-YBX-1, and 1 μg/uL ov-NC using Lipofectamine™ 2000 (Invitrogen) according to the manufacturer’s instructions.

### Tube formation assay

Matrigel (300 mL per well) was plated onto the bottom of six-well plates and incubated at 37 °C for 30 min. Fibroblasts (1 × 10^5^ cells per well) were seeded on Matrigel and induced by EPC-derived exosomes. After a 48-h culture, tube formation was assessed using an inverted microscope (Olympus, Tokyo, Japan).

### Western blotting assay

Western blotting was performed to analyze the expression of CD31, α-SMA, VE-cadherin, vWF, N-cadherin, vimentin, collagen I, YBX-1, SYNCRIP, and hnRNPA2B1 [5]. Specific primary antibodies against CD31 (MA1-81051, 1:100), α-SMA (14-9760-82, 1:500), VE-cadherin (MA5-17050, 1:500), vWF (PA5-80223, 1:1000), N-cadherin (33-3900, 1:1000), vimentin (MA3-745, 1:1000), collagen I (PA1-26204, 1:5000), YBX-1 (PA5-83493, 1:100), SYNCRIP (PA5-50986, 1:500), and hnRNPA2B1 (PA5-34939, 1:5000) were purchased from eBioscience (San Diego, CA, USA), and an HRP-conjugated goat anti-rabbit IgG H&L secondary antibody (1:10000; Southern Biotech, Birmingham, AL, USA) was used. Protein bands were visualized using ECL (Thermo Fisher Scientific). Protein expression was normalized relative to GAPDH expression.

## Results

### miR-133 expression is upregulated in H/R-induced EPC (H/R-EPC)-derived exosomes (H/R-EPC-exosomes)

**Fig. 1:**
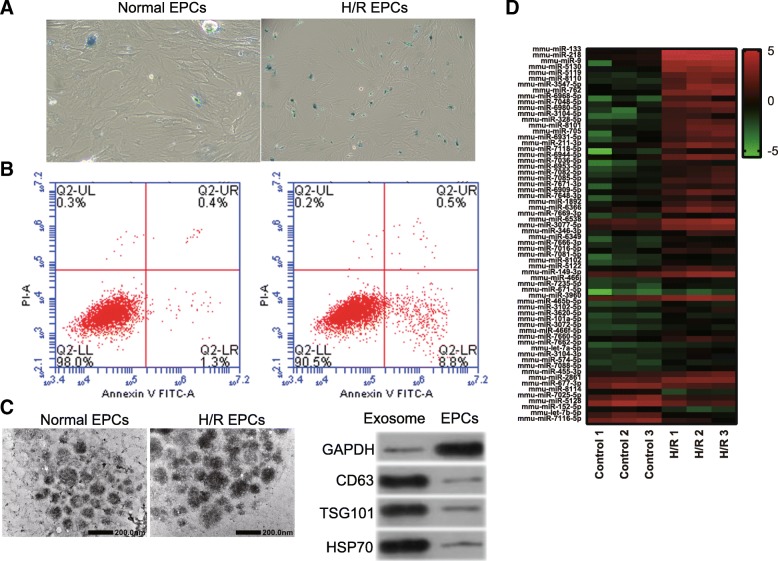
miRNA expression in H/R-induced EPC-derived exosomes. **a** Senescence of H/R-treated EPCs was measured by senescence β-galactosidase staining kit. Senescence cells were stained aquamarine green. **b** Apoptosis of H/R-treated EPCs was measured by the Annexin V-PI Apoptosis Detection Kit. **c** Representative electron microscopy images of exosomes secreted by EPCs. Scale bar, 200 nm. Detection of exosome-associated proteins (including CD63, TSG101, and HSP70) by western blot analysis. **d** Heat map showing the miRNA expression profile as measured by Affymetrix miRNA 4.0 Arrays

**Fig. 2:**
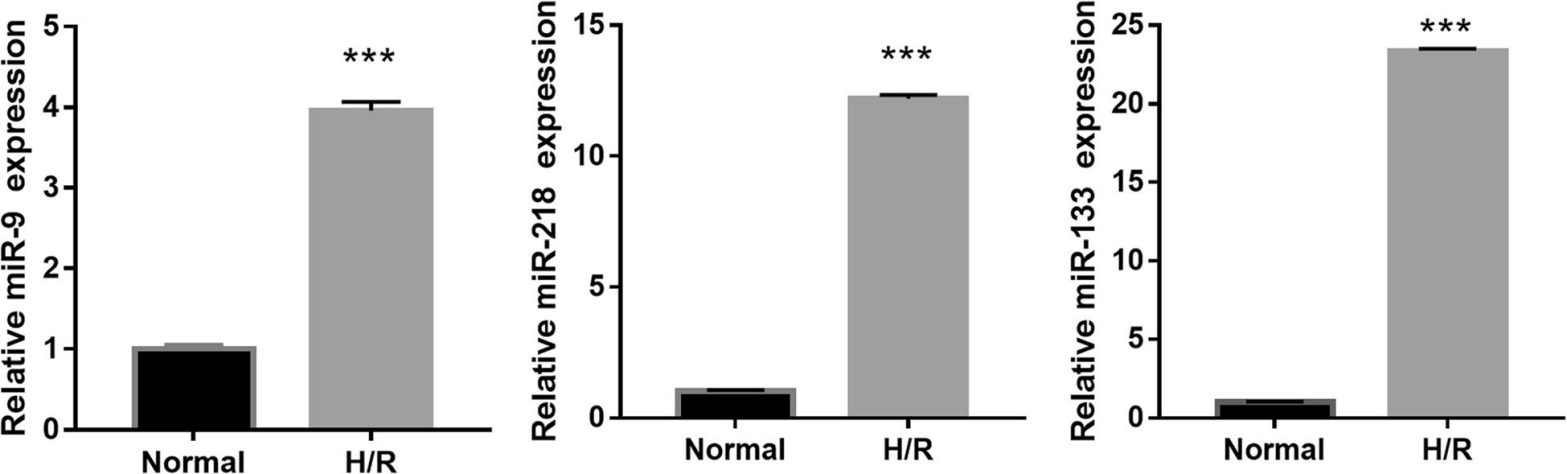
Upregulation of miR-133, miR-218, and miR-9 expression in H/R-induced EPC-derived exosomes. Expression levels of miR-133, miR-218, and miR-9 were measured by qRT-PCR in H/R-induced and normal cultured EPC-derived exosomes. Data are shown as mean ± SD. ***P < 0.001

First, we found that the senescence and apoptosis in H/R-induced EPC were obviously enhanced compared to normal cultured EPCs (Fig. 1a, b). Then, exosomes were purified from culture medium and confirmed by transmission electron microscopy and exosome markers CD63, TSG101, and HSP70 (Fig. 1c). Additionally, miRNA profiles of exosomes from H/R-induced and normal cultured EPCs were investigated using Affymetrix miRNA 4.0 Arrays. The microarray results showed differential expression of 64 miRNAs (7 downregulated miRNAs and 57 upregulated miRNAs, log-fold change > 1; *P* < 0.05) in H/R-EPC-exosomes compared to normal cultured EPC-derived exosomes (Normal-EPC-exosomes) (Fig. 1d). Among 64 miRNAs, only three miRNAs, including miR-133, miR-218, and miR-9, were expressed at greater than five times log-fold change (log-fold change > 5; *P* < 0.05). To validate the accuracy of the microarray assay, expression levels of miR-133, miR-218, and miR-9 were measured by qRT-PCR in H/R-EPC-exosomes and Normal-EPC-exosomes. The results showed that the expression levels of miR-218, miR-9, and especially miR-133 were significantly upregulated in H/R-EPC-exosomes compared to Normal-EPC-exosomes (Fig. 2). Therefore, we focused on miR-133 for further experiments because miR-133 expression had more significant change than miR-218 and miR-9 expression in H/R-EPC-exosomes.

### Intercellular transfer of miR-133 by H/R-EPC-exosomes inhibits fibroblast angiogenesis and MEndoT

**Fig. 3:**
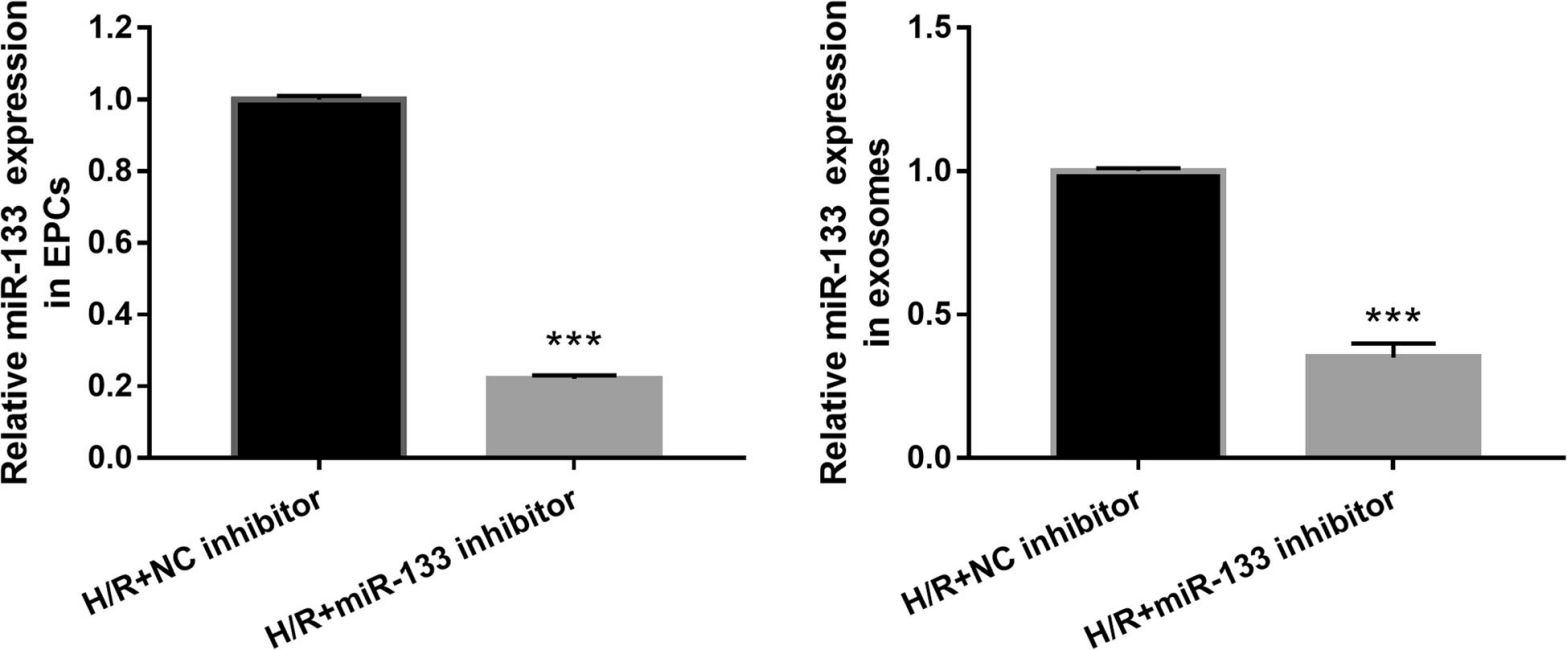
Significant inhibition of miR-133 expression by miR-133 inhibitor transfection in H/R-induced EPCs and H/R-induced EPC-derived exosomes. miR-133 expression was measured by qRT-PCR after transfection with an NC inhibitor and miR-133 inhibitor at 48 h. Data are shown as mean ± SD. ***P < 0.001

**Fig. 4:**
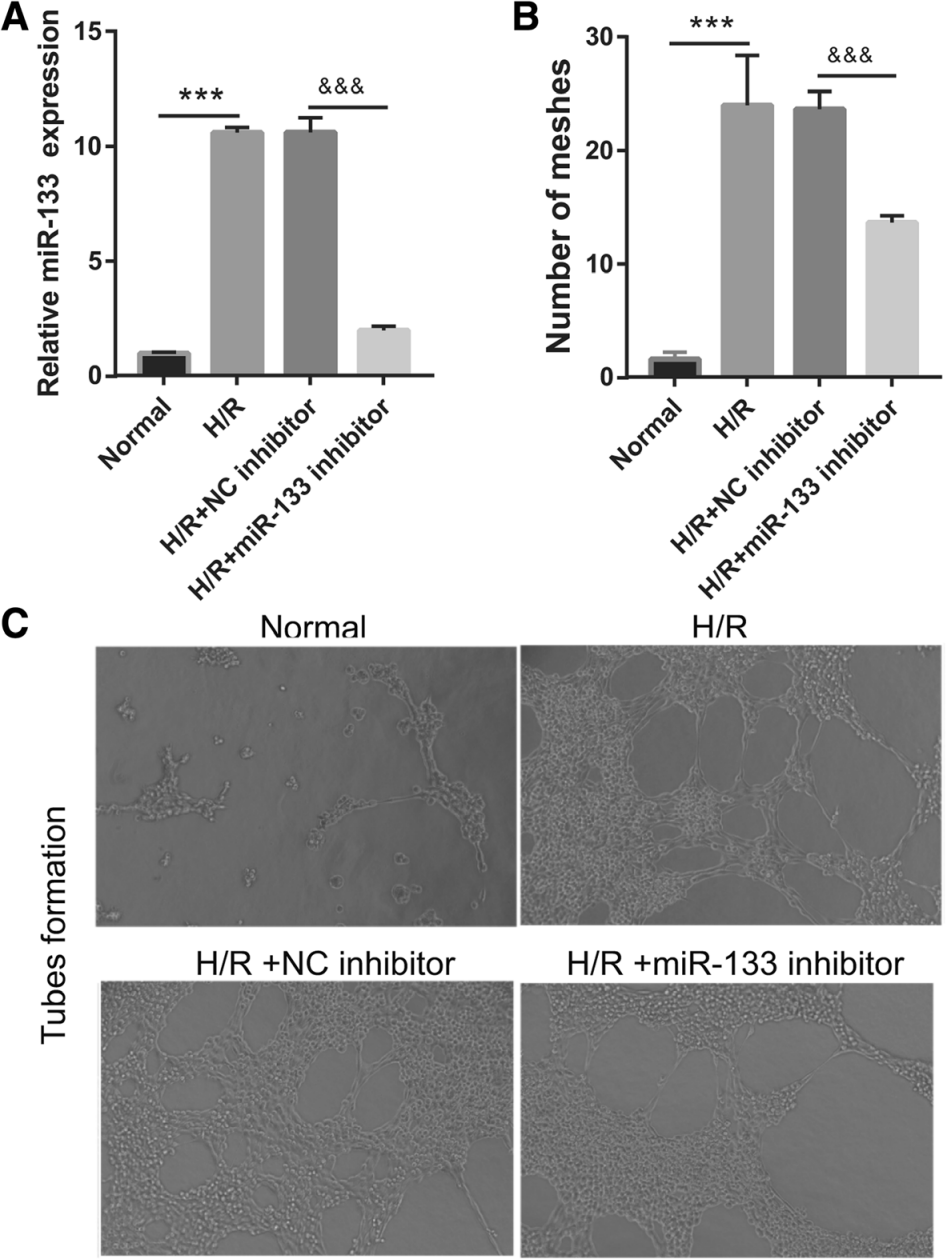
Silencing of miR-133 in H/R-induced EPCs inhibits fibroblast angiogenesis. **a** miR-133 expression was measured by qRT-PCR in fibroblasts treated with EPC-derived exosomes for 48 h. **b** The bar graph represents quantification of the number of meshes per group. **c** Representative image of tube formation analysis. Data are shown as mean ± SD. ***P < 0.001, ^&&&^*P* < 0.001

**Fig. 5:**
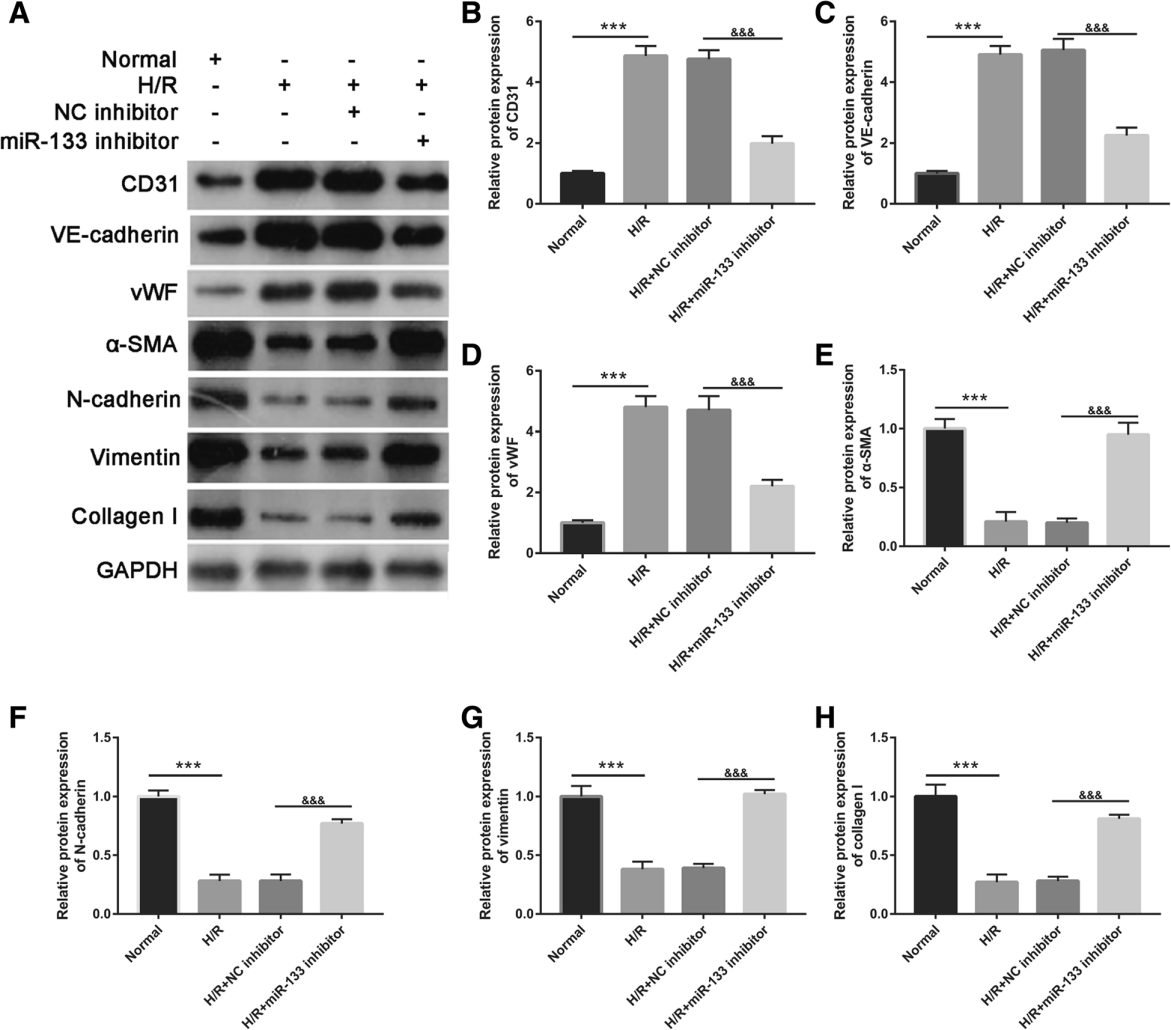
Intercellular transfer of miR-133 by H/R-induced EPC-derived exosomes inhibits fibroblast MEndoT. **a** Endothelial markers CD31, VE-cadherin, and vWF and fibrosis markers α-SMA, N-cadherin, vimentin, and collagen I were measured by western blotting in fibroblasts treated with normal cultured EPC-derived exosomes, H/R-induced EPC-derived exosomes, H/R+NC inhibitor-induced EPC-derived exosomes, and H/R+miR-133 inhibitor-induced EPC-derived exosomes. **b**–**h** The bar graph represents quantification of endothelial markers CD31 (**b**), VE-cadherin (**c**), and Vwf (**d**) and fibrosis markers α-SMA (**e**), N-cadherin (**f**), vimentin (**g**), and collagen I (**h**) expression per group. ***P < 0.001, ^&&&^*P* < 0.001

In an attempt to identify miR-133 required for fibroblast angiogenesis and MEndoT, the miR-133 inhibitor was transfected into H/R-EPC (H/R- miR-133 inhibitor/EPC). The qRT-PCR results showed that miR-133 expression was significantly inhibited after transfection of the miR-133 inhibitor at 48 h in H/R-EPC and H/R-EPC-exosomes compared to NC inhibitor transfection (Fig. 3). Additionally, fibroblasts were treated with H/R-miR-133 inhibitor/EPC-exosomes for 48 h. In the following, angiogenesis and MEndoT of fibroblasts were measured. First, miR-133 expression in fibroblasts treated with Normal-EPC-exosomes was significantly lower than that in fibroblasts treated with H/R-EPC-exosomes and H/R-NC inhibitor/EPC-exosomes. Further, miR-133 expression in fibroblasts treated with H/R-NC inhibitor/EPC-exosomes was significantly higher than that in fibroblasts treated with H/R-miR-133 inhibitor/EPC-exosomes (Fig. 4a). Second, the number of meshes had a similar trend of miR-133 expression in each group, which showed upregulation and downregulation of the number of meshes by miR-133 overexpression and miR-133 knockdown, respectively (Fig. 4b, c). Finally, expression of endothelial markers CD31, VE-cadherin, and vWF in fibroblasts treated with H/R-EPC-exosomes and H/R-NC inhibitor/EPC-exosomes was significantly higher than that in fibroblasts treated with Normal-EPC-exosomes and H/R-miR-133 inhibitor/EPC-exosomes. Conversely, fibrosis markers α-SMA, N-cadherin, vimentin, and collagen I were significantly lower (Fig. 5). The results showed that intercellular transfer of miR-133 by H/R-EPC-exosomes promoted fibroblast angiogenesis and MEndoT.

### YBX-1 expression is upregulated in H/R-EPC

**Fig. 6:**
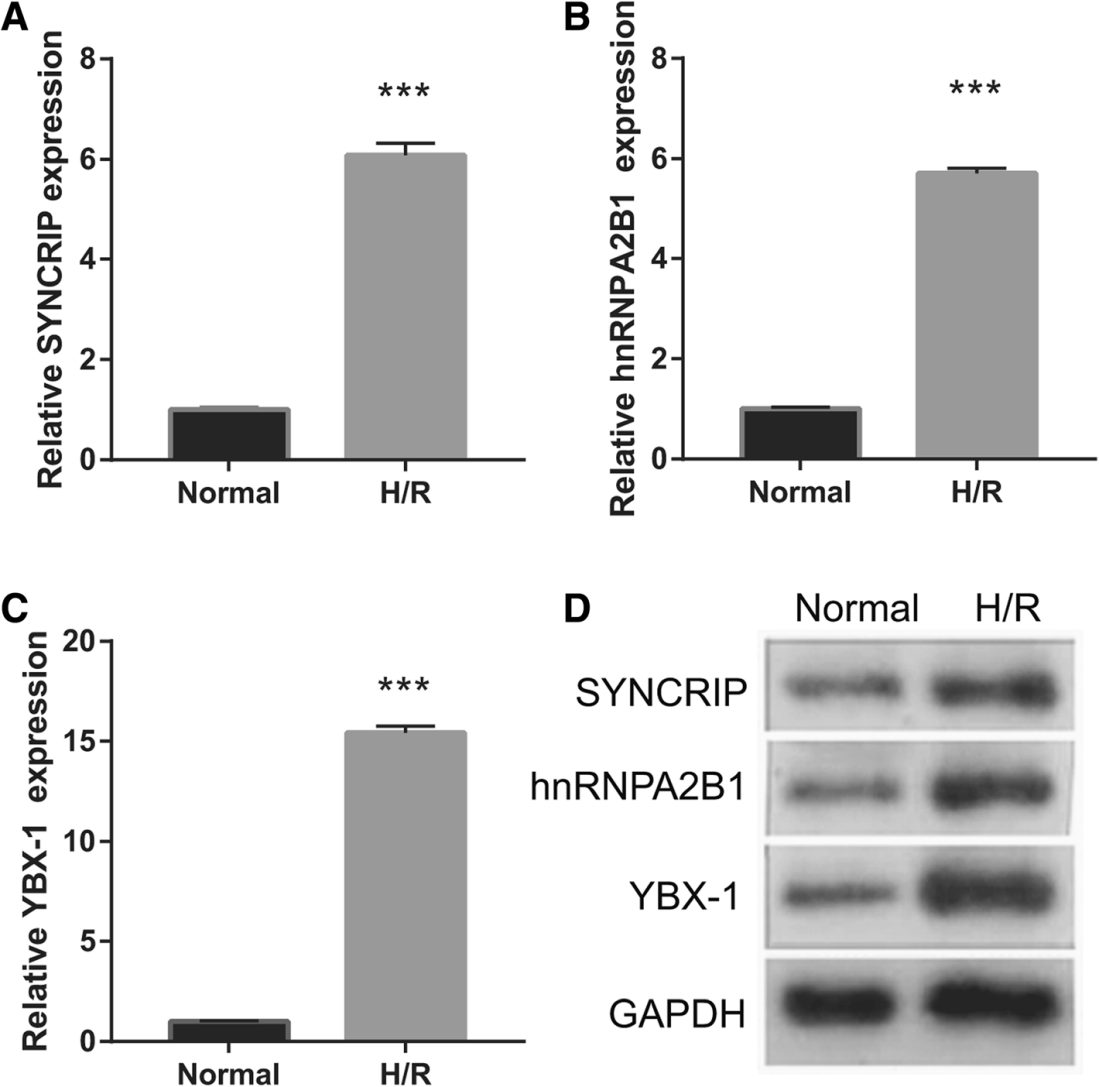
Measurements of YBX-1, SYNCRIP, and hnRNPA2B1 in EPCs by qRT-PCR (**a**–**c**) and western blotting (**d**) after 48-h H/R treatment

We next examined the specific packaging of miR-133 into EPC-derived exosomes, and three reported sorting protein of exosomes, including YBX-1, SYNCRIP, and hnRNPA2B1, in EPCs were measured by qRT-PCR and western blotting. The results showed that YBX-1, SYNCRIP, and hnRNPA2B1 expression levels, especially YBX-1, were significantly upregulated in H/R-EPC compared to Normal-EPC (Fig. 6). Therefore, we focused on YBX-1 for further experiments.

### Packaging of miR-133 into EPC-derived exosomes is mediated by YBP1

**Fig. 7:**
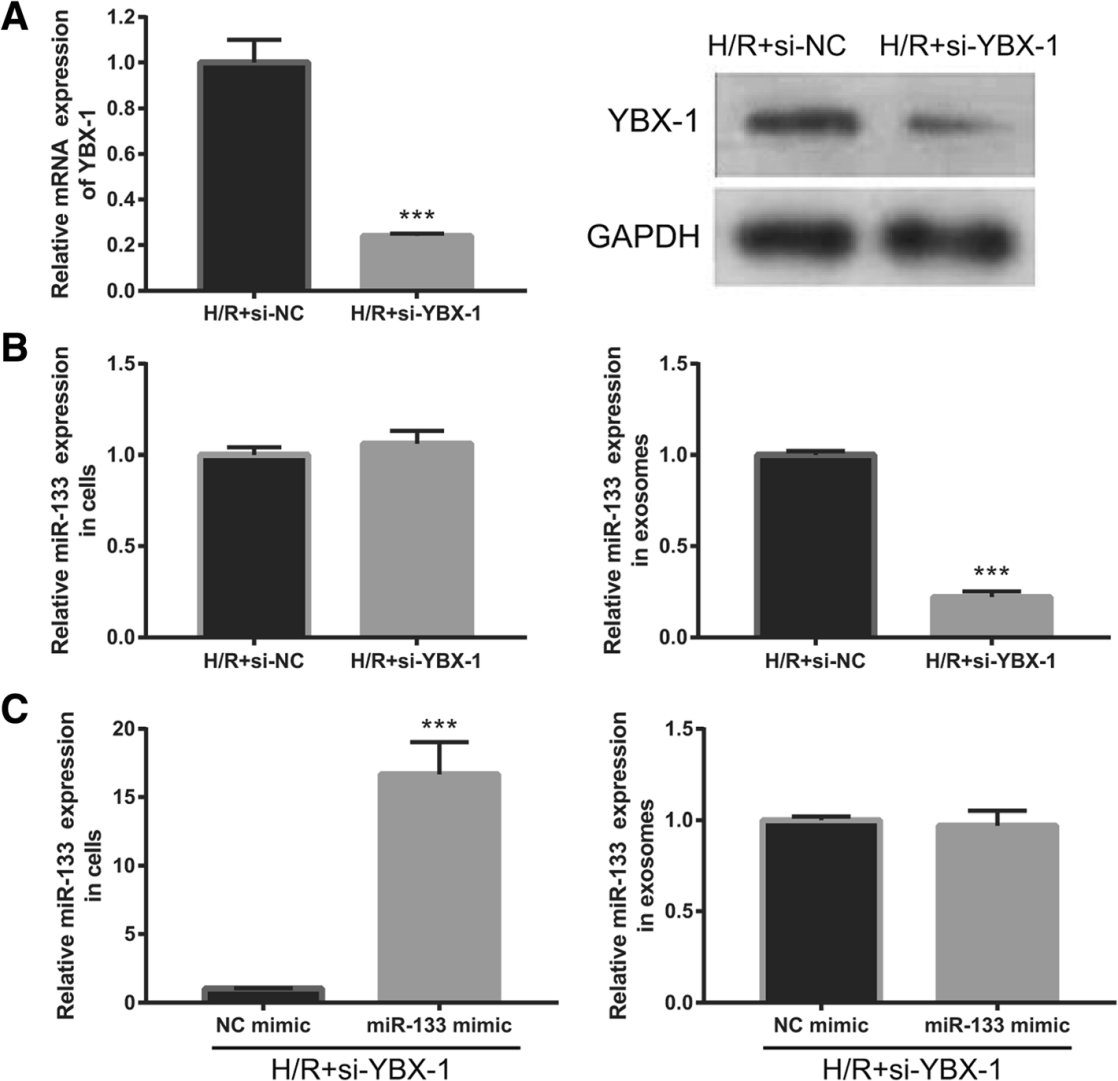
Inhibition of specifically packaged miR-133 into EPC-derived exosomes by YBP1 silencing. **a** qRT-PCR and western blot analysis of YBX1 expression in H/R-induced EPCs at 48 h following si-YBX1 transfection. **b** qRT-PCR analysis of miR-133 expression in H/R-induced EPCs and exosomes at 48 h following si-YBX1 transfection. **c** qRT-PCR analysis of miR-133 expression in H/R-induced EPCs and exosomes at 48 h after co-transfection with si-YBX1 and miR-133 mimic

**Fig. 8:**
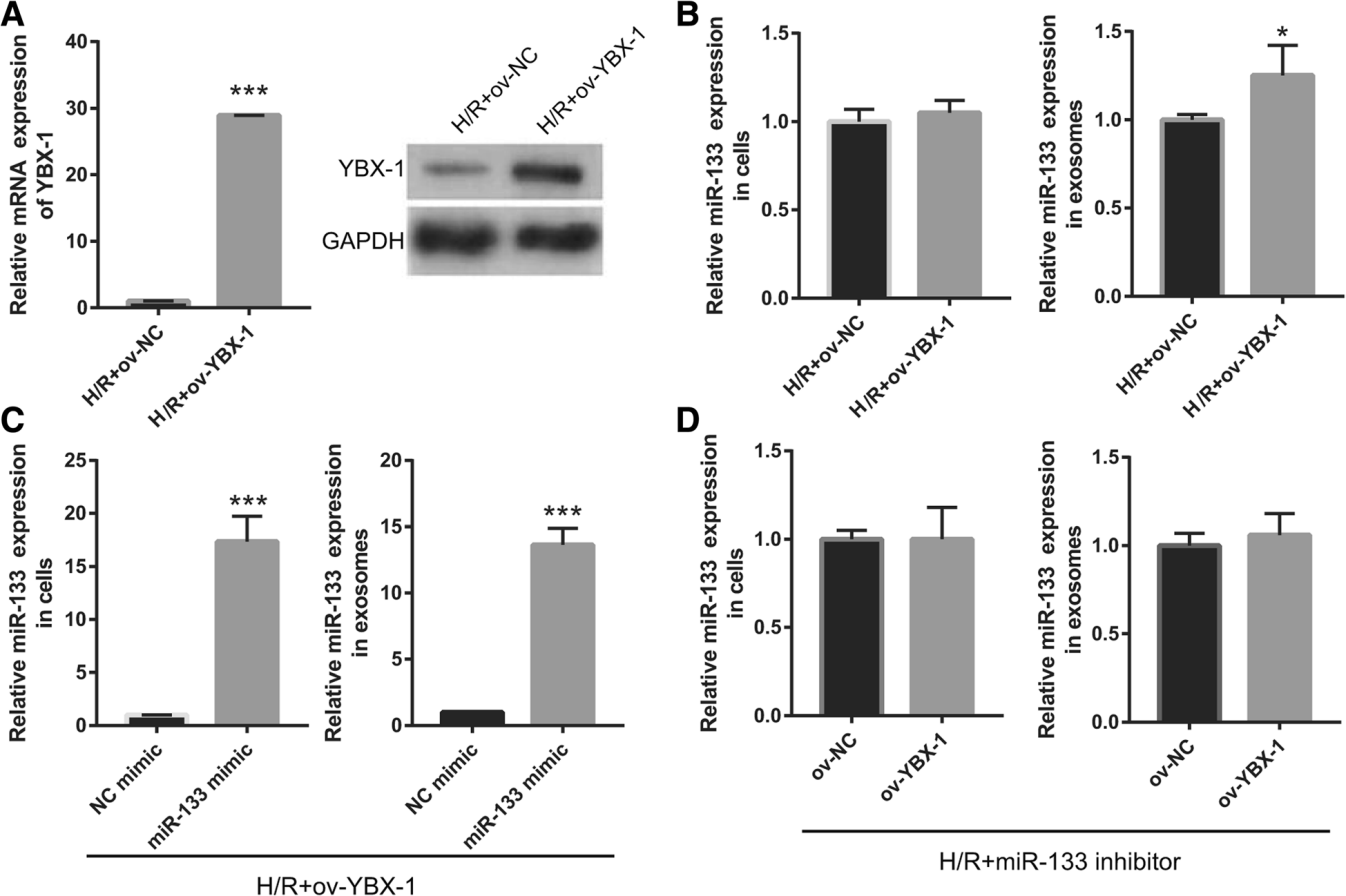
Promotion of specifically packaged miR-133 into EPC-derived exosomes by YBP1 overexpression. **a** qRT-PCR and western blot analysis of YBX1 expression in H/R-induced EPCs at 48 h following ov-YBX1 transfection. **b** qRT-PCR analysis of miR-133 expression in H/R-induced EPCs and exosomes at 48 h following ov-YBX1 transfection. **c** qRT-PCR analysis of miR-133 expression in H/R-induced EPCs and exosomes at 48 h after co-transfection with ov-YBX1 and miR-133 mimic. **d** qRT-PCR analysis of miR-133 expression in H/R-induced EPCs and exosomes at 48 h after co-transfection with ov-NC and miR-133 inhibitor or ov-YBX1 and miR-133 inhibitor

Additionally, we silenced YBX-1 by si-YBX1 transfection to study whether miR-133 was specifically packaged into exosomes by YBP1. YBX1 expression was successfully reduced by si-YBX1 transfection in H/R-EPC (Fig. 7a). As expected, knockdown of YBX1 in H/R-EPC significantly decreased miR-133 expression in H/R-EPC-exosomes; however, it did not change miR-133 expression in H/R-EPC (Fig. 7b). Co-transfected si-YBX1 and miR-133 mimic significantly increased miR-133 expression in H/R-EPC with no effect on miR-133 expression in H/R-EPC-exosomes (Fig. 7c). Next, we upregulated YBX-1 expression by ov-YBX1 transfection to further study whether miR-133 was specifically packaged into exosomes by YBP1. YBX1 expression was successfully enhanced by ov-YBX1 transfection in H/R-EPC (Fig. 8a). As expected, YBX1 overexpression in H/R-EPC had no significant changes in miR-133 expression in H/R-EPC and H/R-EPC-exosomes (Fig. 8b). Co-transfected ov-YBX1 and miR-133 mimic significantly increased miR-133 expression in H/R-EPC and H/R-EPC-exosomes (Fig. 8c). Additionally, ov-YBX-1 transfected had no significant change in the miR-133 expression in the H/R-miR-133 inhibitor-treated EPC cells and exosomes compared to ov-NC transfected (Fig. 8d). These results showed that YBP1 mediated miR-133 packaging into EPC-derived exosomes.

### YBX-1 regulates fibroblast angiogenesis and MEndoT through exosome-transferred miR-133 regulation

**Fig. 9:**
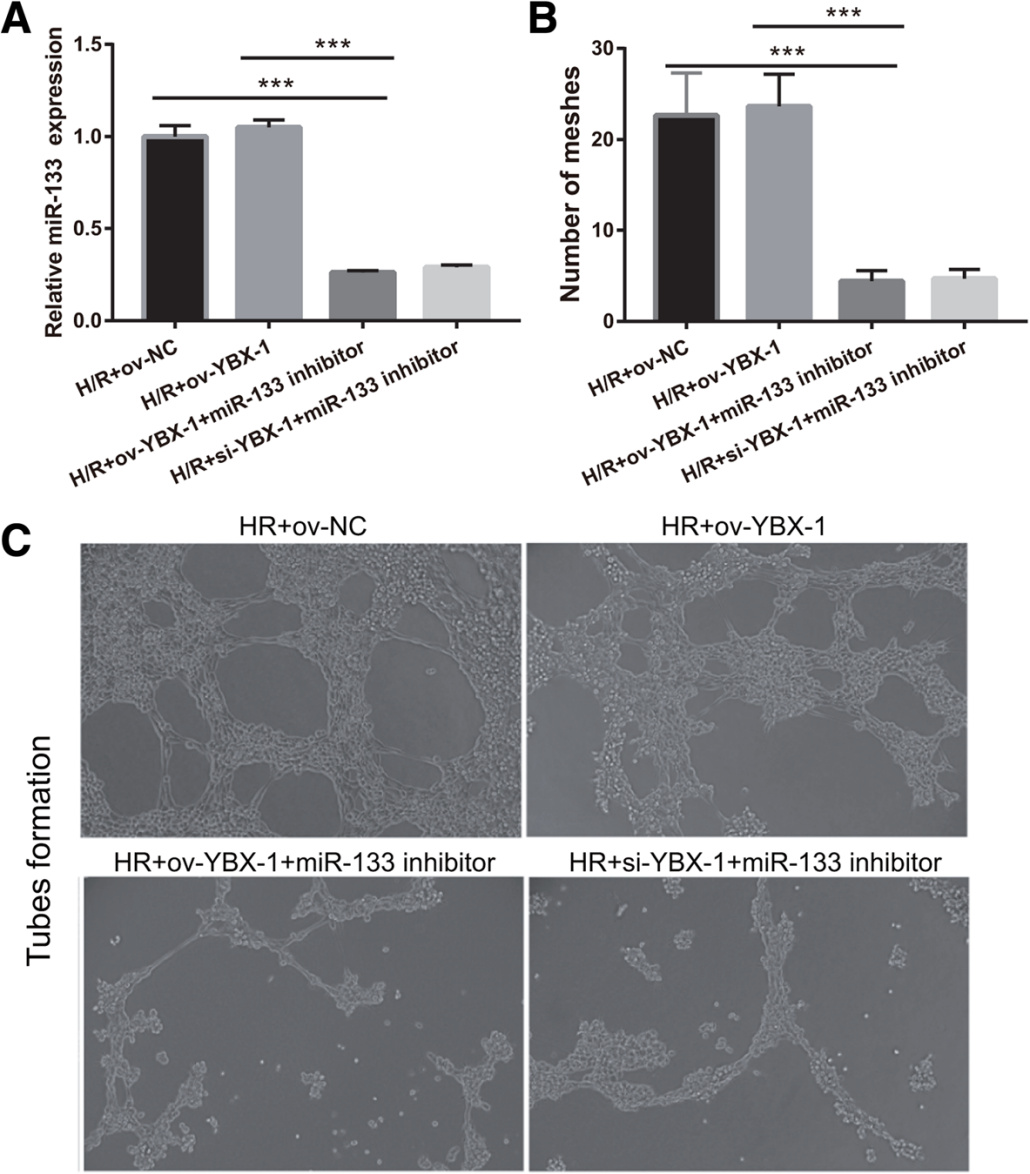
Silencing of YBX1 in H/R-induced EPCs inhibits fibroblast angiogenesis. **a** miR-133 expression was measured by qRT-PCR in fibroblasts treated with H/R, H/R+ov-NC, H/R+si-YBX1+miR-133 inhibitor, and H/R+ ov-YBX1+miR-133 inhibitor-induced EPC-derived exosomes for 48 h. **b** The bar graph represents quantification of the number of meshes per group. **c** Representative image of tube formation analysis. Data are shown as mean ± SD. ***P < 0.001

**Fig. 10:**
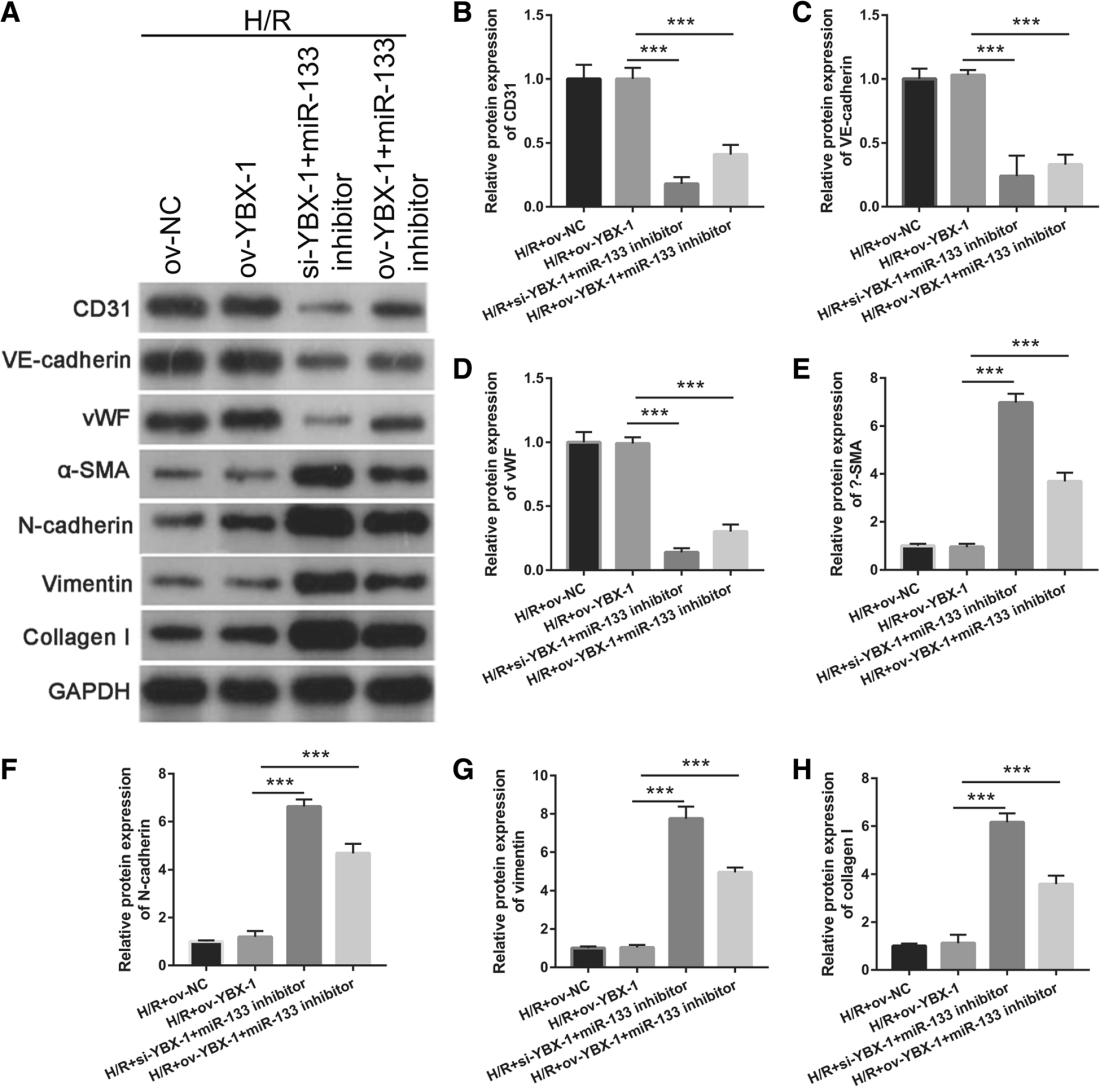
Intercellular transfer of miR-133 by H/R-induced EPC-derived exosomes inhibits fibroblast MEndoT. **a** Endothelial markers CD31, VE-cadherin, and vWF and fibrosis markers α-SMA, N-cadherin, vimentin, and collagen I were measured by western blotting in fibroblasts treated with H/R, H/R+ov-NC, H/R+si-YBX1+miR-133 inhibitor, and H/R+ ov-YBX1+miR-133 inhibitor-induced EPC-derived exosomes. **b**–**h** The bar graph represents quantification of endothelial markers CD31 (**b**), VE-cadherin (**c**), and vWF (**d**) and fibrosis markers α-SMA (**e**), N-cadherin (**f**), vimentin (**g**), and collagen I (**h**) expression per group. ***P < 0.001

We further examined whether YBX-1 could regulate miR-133 transfer to reduce fibroblast angiogenesis and MEndoT. First, miR-133 expression in fibroblasts treated with H/R-si-YBX1+miR-133 inhibitor/EPC-exosomes and H/R-ov-YBX1+miR-133 inhibitor/EPC-exosomes was significantly lower than that in fibroblasts treated with H/R-ov-YBX1/EPC-exosomes and H/R+ov-NC/EPC-exosomes (Fig. 9a). The number of meshes had a similar trend of miR-133 expression in each group (Fig. 9b, c). Finally, endothelial markers CD31, VE-cadherin, and vWF expression levels in fibroblasts treated with H/R-si-YBX1+miR-133 inhibitor/EPC-exosomes and H/R-ov-YBX1+miR-133 inhibitor/EPC-exosomes were significantly lower than fibroblasts treated with H/R-ov-YBX1/EPC-exosomes and H/R+ov-NC/EPC-exosomes. In contrast, fibrosis markers α-SMA, N-cadherin, vimentin, and collagen I were significantly higher (Fig. 10). The results showed that fibroblasts treated with H/R-ov-YBX1/EPC-exosomes and H/R+ov-NC/EPC-exosomes had no significant effects on fibroblast angiogenesis and MEndoT. Fibroblast angiogenesis and MEndoT were significantly inhibited in fibroblasts treated with H/R-si-YBX1+miR-133 inhibitor/EPC-exosomes and H/R-ov-YBX1+miR-133 inhibitor/EPC-exosomes compared to fibroblasts treated with H/R-ov-YBX1/EPC-exosomes due to miR-133 silencing. The results suggested that YBX-1 knockdown inhibited miR-133 exosome transfer to reduce fibroblast angiogenesis and MEndoT.

## Discussion

Cardiovascular disease induces fibroblast proliferation and excessive activation, resulting in cardiac fibrosis. Fibroblasts undergo MEndoT to obtain endothelial cell-like functions and participate in angiogenesis in the cardiac injury area, which can reverse myocardial fibrosis [4]. In a previous study, we found that H/R-EPC-exosomes increase angiogenesis in cardiac fibroblasts by promoting MEndoT. Hence, it is necessary to study the mechanism of H/R-EPC-exosomes to promote conversion of cardiac fibroblasts into endothelial cells and identify novel targets for cardiac fibrosis therapy. In this study, we found that H/R-EPC-exosomes increased angiogenesis in cardiac fibroblasts by promoting MEndoT. Additionally, miR-133 was overexpressed in H/R-EPC-exosomes and functionally required for MEndoT of cardiac fibroblasts.

EPCs, a type of hematopoietic stem cell, promote vascular repair, including vascular proliferation and remodeling, when organs suffer from ischemic injury or endothelial damage [22, 23]. EPCs have been identified to participate in the alleviation of cardiac fibrosis in several cardiovascular diseases [24, 25]. Several studies have described effective EPC-mediated attenuation of renal/fibrosis via exosomes [5, 26]. EPC-derived exosomes are complex particles formed by exocytosis of the EPC cell membrane, which can act as effective messengers for transmission of cell signaling and biological function between cells [5, 27]. Importantly, we found that H/R-induced EPC-derived exosomes increased angiogenesis in cardiac fibroblasts by promoting MEndoT [5]. Here, we studied the contribution of exosomal miRNAs in regulating the MEndoT of cardiac fibroblasts. Our results found that H/R treatment promoted the senescence and apoptosis of EPCs. Wang et al. found that serum deprivation plus TNFα stimulation promoted EPC apoptosis and exosome release to improve H/R-induced endothelial dysfunction [28]. These results suggested that the onset of senescence or apoptosis may greatly affect the release of exosomes. Additionally, miR-133 was overexpressed in H/R-EPC-exosomes by a miRNA microarray assay and qRT-PCR verification. H/R-EPC-exosome treatment inhibited promoted fibroblast angiogenesis and MEndoT. We also found that silencing of miR-133 expression in EPCs inhibited miR-133 expression in EPC-derived exosomes, which inhibited the intercellular transfer of miR-133 into fibroblasts and inhibited fibroblast angiogenesis and MEndoT. The results suggested that EPC-derived exosomes promoted fibroblast angiogenesis and MEndoT through the intercellular transfer of miR-133 thereby attenuating myocardial fibrosis.

Studies have shown that the mechanism by which miRNAs are specifically loaded into exosomes is associated with specific RNA-binding proteins, such as YBX-1 [29], SYNCRIP [30, 31], and hnRNPA2B1 [32]. Here, we uncovered that YBX-1 expression was significantly enhanced in H/R-EPC as compared to SYNCRIP and hnRNPA2B1 expression. YBX1, which is localized to cytoplasmic granules, is an RNA-/DNA-binding multifunctional protein. The YBX1 protein is one of the most important transcriptional regulator proteins in exosomes [33]. Recently, some studies have found that Y box protein 1 is required to sort mRNAs, miRNAs, and lncRNA into exosomes [29, 34–36]. In this study, we found that YBX-1 upregulation in H/R-EPC did not affect miR-133 expression in H/R-EPC and H/R-EPC-exosomes. Additionally, YBX-1 upregulation did not affect fibroblast angiogenesis and MEndoT. Furthermore, we found that YBX-1 silencing did not affect miR-133 expression in H/R-EPC; however, it inhibited miR-133 expression in H/R-EPC-exosomes and prevented fibroblast angiogenesis and MEndoT. These results suggested that miR-133 was specially sorted into H/R-EPC-exosomes via YBX-1. Further, YBX-1 silencing inhibited miR-133 transfer and reduced fibroblast angiogenesis and MEndoT.

## Conclusion

miR-133 was specially sorted into H/R-EPC-exosomes via YBX-1 to increase fibroblast angiogenesis and MEndoT. Our findings suggest that miR-133 and YBX-1 are potential therapeutic targets to improve myocardial fibrosis.

## Data Availability

The datasets generated and/or analyzed during the current study are available from the corresponding author on reasonable request.

## Abbreviations

EPC: Endothelial progenitor cell
MEndoT: Mesenchymal-endothelial transition
H/R: Hypoxia/reoxygenation
YBX-1: Y box binding protein 1
H/R-EPC: H/R-induced EPC
H/R-EPC-exosomes: H/R-EPC-derived exosomes
Normal-EPC-exosomes: Normal cultured EPC-derived exosomes
